# Understanding the exodus: a 15-year retrospective cohort study on the pattern and determinants of migration among Nigerian doctors and dentists

**DOI:** 10.1080/16549716.2024.2432754

**Published:** 2024-11-29

**Authors:** Oghenebrume Wariri, Patience Toyin-Thomas, Itua C.G. Akhirevbulu, Oladapo Oladeinde, Oluchi Omogbai, Philippa Odika, John Osakue, Avwebo Ukueku, Efetobo Orikpete, Chinelo Iwegim, Efe E. Omoyibo, Jermaine Okpere, Uwaila Otakhoigbogie, Ekhosuehi T. Agho, Sunday C. Madubueze, Nnennaya C. Ugoji, Chukwunwike W. Ozegbe, Oti N. Aria, Paul Ikhurionan

**Affiliations:** a Department of Infectious Disease Epidemiology, London School of Hygiene and Tropical Medicine, London, UK; b Vaccines and Immunity Theme, Medical Research Council Unit The Gambia at London School of Hygiene and Tropical Medicine, Atlantic Boulevard, Fajara, The Gambia; c The Department of Pediatrics, Geisel School of Medicine, Dartmouth College, Hanover, New Hampshire, USA; d The Dartmouth Institute for Health Policy and Clinical Practice, Lebanon, New Hampshire, USA; e Orthopaedics and Traumatology Division, Department of Surgery, Edo Specialist Hospital, Benin City, Nigeria; f Department of Family Medicine, Medicine Hat Regional Hospital, Medicine Hat, Alberta, Canada; g Independent Researcher, Abbotsford, British Columbia, Canada; h Department of Public Health, Faculty of Health, Education and Society, University of Northampton, UK; i Department of Anaesthetics, Hull University Teaching Hospital NHS Trust, Hull, England, UK; j Department of Obstetrics and Gynaecology, University of Port Harcourt Teaching Hospital, Port Harcourt, Nigeria; k Department of Oral Pathology, Oral Medicine and Oral Radiology, Faculty of Dentistry University of Port Harcourt, Nigeria; l Medical Microbiology Department, University of Toronto, Toronto, Canada; m Department of Paediatrics, Federal Medical Centre, Asaba, Nigeria; n Tom Baker Cancer Centre, Alberta Health Services, Calgary, Alberta, Canada; o Department of Oral Pathology and Oral Medicine, Faculty of Dentistry, College of Medicine, Ituku/Ozalla Campus, University of Nigeria, Nigeria; p Dental and Maxillofacial Surgery Department, National Hospital Abuja, Nigeria; q Department of Family Medicine, Dalhatu Araf Specialist Hospital (DASH), Lafia, Nigeria; r School of Paediatrics, Health Education, North East, England, UK; s Department, Obstetrics and Gynaecology, Model Hospital, Akwanga, Nigeria; t Burns, Plastic and Reconstructive Surgery Unit, Department of Surgery, Rivers State University Teaching Hospital, Port Harcourt, Nigeria; u Department of Child Health, University of Benin Teaching Hospital, Benin-city, Nigeria

**Keywords:** Migration, human resource for health, doctor, Nigeria, retrospective cohort

## Abstract

**Background:**

Nigeria faces a critical shortage of healthcare professionals yet experiences a significant annual exodus of doctors and dentists. This alarming trend threatens the country’s ability to provide equitable healthcare.

**Objective:**

This study investigated the patterns and determinants of migration among doctors and dentists who graduated from the University of Benin, Nigeria, 15 years ago.

**Methods:**

We conducted a retrospective cohort study that tracked 274 of the 379 (72.3%) eligible cohort. We computed the migration incidence rate per person-year from 2008 to 2023, covering 3,455 person-years of follow-up and analysed migration drivers as push and pull factors across macro-, meso-, and micro-levels.

**Results:**

Fifteen years post-graduation, 48.9% (134/274) of the cohort had migrated. While the annual incidence rate of migration remained stable for the first 8 years, it spiked after 2016, reaching 11.4 per 100 person-years in 2023. Among those who migrated, the majority (96.3%, 129/134) relocated outside the African continent. The top three destination countries were the UK (48.5%, 65/134), Canada (20.9%, 28/134), and the USA (19.4%, 26/134). The leading push factors were insecurity of lives and property (57.8%), concerns about children’s futures (50.3%), and limited career development opportunities (45.9%). The primary pull factors included security (56.3%), permanent residency (49.6%), and better pay in the destination country (46.7%). Significant predictors of migration included younger age, timing of marriage, and residency training status.

**Conclusions:**

To avert an impending crisis, the Nigerian government must address the root causes driving the increasing migration of doctors and dentists.

## PAPER CONTEXT

**Main findings:**

Within a cohort of Nigerian doctors and dentists, nearly half emigrated within 15 years, with migration rates accelerating after 2016 due to insecurity, low pay, and limited career prospects.

**Added knowledge:**

This is the first cohort study in sub-Saharan Africa to document the temporal pattern and specific drivers of migration among doctors and dentists, underscoring the impact of professional and economic instability on health workforce retention.

**Global health impact on policy and action:**

Addressing the underlying causes of doctor and dentist migration is crucial for reducing health disparities and advancing universal health coverage in low- and middle-income countries.

## Background

The migration of the health workforce is a significant global health issue with far-reaching implications for both the source and destination countries [1,2]. In source countries, the outflow of skilled health workforce exacerbates existing shortages, overwhelms healthcare infrastructures, and hinders health system sustainability [3]. On the other hand, in destination countries, the migrating health workforce makes valuable contributions by compensating for local personnel shortages and improving the quality of care that can be provided [4]. Many high-income countries (HICs) face persistent shortages of domestically trained health workforce, leading to increased reliance on foreign-trained personnel, often from low-and middle-income countries (LMICs), to operate effectively [4–6]. The World Health Organization (WHO) has consistently highlighted critical health workforce shortages across various LMIC settings in Africa, the Middle East, and Asia [7]. The WHO emphasises that these countries struggle to deliver essential health services, often with fewer than 23 doctors, nurses, and midwives per 10,000 population [8]. The economic impact of health workforce migration on LMICs can be devastating. These countries incur an estimated annual economic loss of US$15.86 billion due to excess mortality associated with doctor migration, with India, Nigeria, Pakistan, and South Africa incurring the most substantial financial impact [9].

The WHO African region has a critical shortage of the health workforce, with approximately two doctors per 10,000 population and 10 nurses or midwives per 10,000 people, compared to the global median of 49 doctors, nurses, and midwives per 10,000 people [10]. Nigeria exemplifies this critical shortage and is among 55 countries included in the 2023 WHO ‘*Health Workforce Support and Safeguards List*’ [10] due to its low health workforce density of 3.9 doctors per 10,000 population [11] and inadequate coverage of essential health services. The countries on this list face the most pressing health workforce challenges needed to achieve universal health coverage and must be prioritised for health workforce support by governments and the international community. These countries should also be safeguarded by discouraging active international recruitment. Despite an already low doctor–population ratio, Nigeria has witnessed a considerable exodus of doctors and dentists over the past decade. By 2021, less than half of its approximately 80,000 registered doctors and dentists were practising domestically [12]. Moreover, in 2022, 500 doctors and dentists left Nigeria for HICs over the preceding 2 years, and nine out of every 10 specialists with less than 5 years of experience plan to leave the country [12]. This alarming trend in brain drain jeopardises the Nigerian health system’s capacity to deliver quality and equitable care.

In a recent systematic review on health workforce migration, encompassing five decades and 107 studies from over 90 LMICs, conducted by our team, we found that key migration drivers operate at macro- (global and national), meso- (professional), and micro- (personal) levels [13]. Nevertheless, important gaps persist in the existing research. Most studies from sub-Saharan Africa have focused on the ‘intention to migrate’; thus, the reported drivers of migration in these studies might potentially differ when ‘actual’ migration is considered specifically. Furthermore, the majority of the studies in the systematic review [13], and recent ones from Nigeria [12,14,15], utilised cross-sectional designs and examined doctors and dentists from diverse backgrounds and contexts, limiting the depth of their findings on the drivers and patterns of migration. Taken together, there is a dearth of longitudinal studies examining the patterns and determinants of doctor and dentist migration in sub-Saharan Africa, which is necessary to guide policy interventions. This underscores the urgent need for more comprehensive and longitudinal research to inform effective policies that address the root causes of doctor and dentist migration in this region.

Cohort studies offer a superior approach to understanding complex and ongoing issues like doctor and dentist migration compared to cross-sectional studies [16]. They provide longitudinal data on migration patterns and determinants, allowing for the examination of how the patterns evolve over time and yielding deeper insights into the complex dynamics of a phenomenon. To address the identified evidence gaps, this study investigated the patterns and determinants of migration among a cohort of medical and dental graduates from the University of Benin, Nigeria, who completed their studies in 2008. Specifically, the study determined the prevalence and incidence of migration and explored the macro-, meso-, and micro-level factors that influenced migration decisions.

## Methods

### Overview of medical training

As of August 2024, Nigeria had 43 fully accredited and 14 partially accredited medical and dental schools, with a combined annual admission quota of approximately 6,500 students [17]. The University of Benin (UNIBEN), a federal institution in South–South Nigeria, was founded in 1970, with its College of Medical Sciences (i.e. the Medical and Dental School) was established in 1973 [18]. UNIBEN Medical School is one of the second-generation medical schools established in Nigeria, after the first-generation institutions in Ibadan, Lagos, Zaria, Enugu, and Ile-Ife [19,20]. It is the oldest medical school in present-day South–South Nigeria, with one of the country’s highest yearly admission quotas at 175 students (i.e. 150 in medicine and 25 in dentistry) [17,21].

The medical school admits qualified candidates from diverse backgrounds, across all regions of Nigeria and other countries who have successfully passed the Joint Admissions and Matriculation Board (JAMB) exams and met other admission criteria for its medical or dental programmes. Both programmes span approximately 6 years, consisting of 3 years of preclinical and 3 years of clinical training. Upon completing all qualification examinations, candidates can graduate with either a Bachelor of Medicine, Bachelor of Surgery (MBBS), or a Bachelor of Dental Surgery (BDS) degree. These newly qualified doctors and dentists undergo a 12-month supervised internship at accredited hospitals, after which they become eligible for full licensure, allowing them to practise independently as medical or dental officers.

Following internship, these doctors and dentists undergo a mandatory National Youth Service Corps (NYSC) for 12 months or are exempted if they are 30 years or older. During this period, they are assigned as medical and dental officers to various hospitals across the country to serve the community. Upon completing their NYSC year, doctors and dentists can pursue specialist training in accredited training hospitals after passing the requisite primary examinations and securing a residency position in their chosen field. Alternatively, they can choose not to pursue specialty training, and instead work in general practice as medical or dental officers in clinical settings or pursue other career paths.

### Study design

This was a retrospective cohort study. For the purpose of this study, migration was operationally defined as the movement of cohort members from Nigeria to another country to live, for employment, or pursue other activities, regardless of the duration they had been abroad at the time of data collection. This definition excludes short-term stays for courses, training programmes, and conferences.

### Study population

This cohort study included all 383 doctors and dentists who graduated from the University of Benin Medical School in 2008. We obtained a verified, senate-approved list of cohort members from the university. Since the list lacked contact details and the university had no formal system for reaching past graduates, we used a snowball approach to gather contact information, creating a database with phone numbers and email addresses. This approach was effective as the research team members were also 2008 graduates with longstanding connections to many cohort members.

To maximise cohort inclusion and ensure equal participation opportunity, we used a modified Delphi process alongside a snowballing approach. All 23 research team members reviewed the official list, identifying contacts within their networks for each cohort member. If no connections were found, the member was deemed untraceable. Four members were deceased, leaving 379 eligible for the study. We successfully obtained contact information for 303 members, of whom 298 agreed to participate and received the study questionnaire.

### Data collection

We collected data using a structured and pretested electronic self-administered questionnaire specifically designed for this study (supplementary appendix). The questionnaire development was guided by evidence from a systematic review on health worker migration covering five decades and 107 studies from over 90 LMICs [13]. The electronic questionnaire was developed using KoboToolbox® [22], a free data collection and management platform.

Participants received an email invitation at the beginning of the study with a unique study number and an embedded link to access the online questionnaire. Data collection occurred over a 7-week period between 3 March and 20 April 2024. To maximise the response rate, we conducted biweekly follow-ups with three email reminders and a final attempt via phone calls in the last week of data collection.

Before data collection began, we conducted a sensitisation exercise through virtual open days on the 2008 University of Benin medical and dental alumni WhatsApp group. Members were informed about the proposed cohort study and had their questions answered. Participation in the study was entirely voluntary, and informed consent was sought electronically and obtained during the recruitment process.

Members of the 2008 cohort who were part of the research team were included as study participants. To ensure ethical standards, minimise bias, and protect data privacy, we implemented several measures. First, only one team member had access to the backend data via KoboToolbox® and managed the data, ensuring the team members’ responses remained confidential. Second, data analysis was conducted by a different team member without access to any identifiable information. Third, the dataset provided for analysis was password-protected, further safeguarding privacy. Lastly, any required clarifications on the dataset were obtained directly from the data manager.

### Data preparation

During data preparation, some continuous variables (e.g. age, family size, etc.) were recategorised into categorical variables. To ensure that these categories were meaningful, we employed a tertile approach. This method statistically divides each continuous variable into three quantiles (tertiles), each containing approximately one-third of the population. This strategy avoids the potential bias of creating arbitrary categories, which could lead to misleading interpretations [23].

The dataset contained missing values in hierarchical variables, such as marital status and postgraduate training details. To address this, a two-pronged approach was adopted. First, we created indicator variables to explicitly acknowledge these missing values within the hierarchical structure, clarifying that the missing information logically does not apply to these respondents. Second, multiple imputation by chained equations (MICE) was implemented using the ‘mice’ package in R to generate the five imputed datasets [24]. The predictive mean matching (PMM) method was employed for imputation.

### Statistical analysis

We calculated the incidence rate of migration per person-year for the entire cohort from 2008 to 2023. This rate represents the number of migration events divided by the total person-years contributed by the population ‘at risk of migration.’ The latter refers to the cumulative person-years of individuals who had not yet migrated by the end of the preceding year. We assigned a contribution of 0.5 person-years per individual for 2008 since the cohort graduated in July of that year. From 2009 to 2023, each person remaining in the population at risk (i.e. not having migrated) contributed 1 person-year.

We examined the push and pull drivers of migration [25], categorised as macro, meso, and micro-level factors based on the conceptual framework established by Young et al. [26]. Push factors refer to the circumstances that influence people to leave their home countries, such as Nigeria, while pull factors are those that attract them to a foreign country [25]. According to Young’s framework, macro-level factors encompass global and national influences, meso-level factors are related to professional considerations, and micro-level factors pertain to personal circumstances that shape migration choices [26].

To analyse the sociodemographic factors associated with migration, we initially fitted bivariate logistic regression models. These initial models examined the unadjusted association of each covariate (independent variable) with migration (dependent variable). Subsequently, we fit multiple logistic regression models to evaluate the associations between migration and individual-level characteristics and obtained adjusted odds ratios (aORs) and 95% confidence intervals (CIs). All analyses were performed using R software (R Development Core Team, 2023) [27].

### Ethics

The study received ethical approval (CMS/REC/2023/457; Date: 23 October 2023) from the Research Ethics Committee, College of Medical Sciences, University of Benin, Edo state, Nigeria.

### Results

#### Characteristics of the cohort

**Figure 1.**
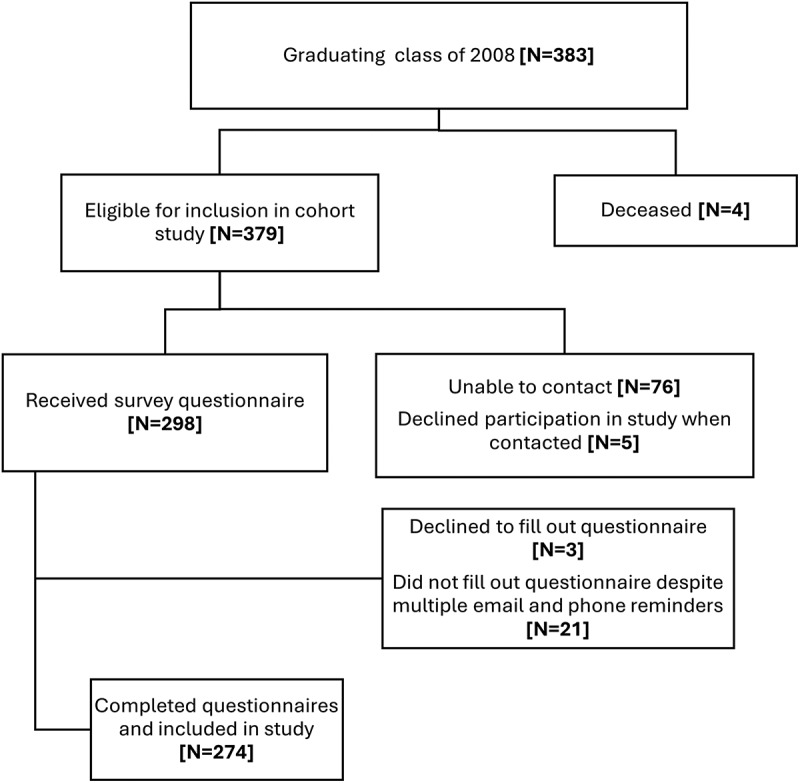
Flowchart illustrating the 2008 graduating cohort, those eligible for inclusion, those who received the study questionnaire and those who completed the study questionnaire.

Out of the eligible cohort, 72.3% (274/379) of the participants completed the questionnaire. The response rate was 70.1% (225/321) among doctors (MBBS) and 84.5% (49/58) among dentists (BDS). By sex, the response rate was 68.5% (174/254) for males and 80% (100/125) for females. Figure 1 provides a detailed flowchart outlining the number of participants who did not complete the questionnaire and those included in the final cohort.

Among the 274 participants, 49 (17.9%) were dentists, closely mirroring the 15.3% (58/379) in the eligible cohort, while 225 (82.1%) were doctors, comparable to 84.7% (321/379) in the eligible cohort. Additionally, 36.5% (100/274) of participants were females, compared to 33% (125/379) in the eligible cohort, while males represented 63.5% (174/274), slightly lower than the 67% (254/379) in the eligible cohort (Table 1).

**Table 1.** Characteristics of 274 doctors and dentists 15 years after graduation from the University of Benin Medical School.

Variable	Dentists (BDS) (*n* = 49)	Doctors (MBBS) (*n* = 225)	Total (*N* = 274)
Age			
Median age (years) Mean age (years) Age range (years)	42 41.8 38–47	41 42.5 38–60	42 42.4 38–60
Sex, n (%)			
Female Male	20 (40.8) 29 (59.2)	80 (35.6) 145 (64.4)	100 (36.5) 174 (63.5)
Marital Status, n (%)			
Married Not Married*	44 (89.8) 5 (10.2)	202 (89.8) 23 (10.2)	246 (89.8) 28 (10.2)
Timing of Marriage			
Median year married Range (year married)	2012 2004 – 2021	2012 1993 – 2021	2012 1993 – 2021
Dependents**			
Nuclear family dependents (median) Nuclear family dependents (range) Non-nuclear family dependents (median) Non-nuclear family dependents (range)	4.5 1–8 2 0–10	5 1–10 2 0–21	5 1–10 2 0–21
Commenced Postgraduate Training, n (%)			
Residency/Fellowship Masters Degree PhD Other Training**	25 (51.0) 18 (36.7) 3 (6.1) 6 (12.2)	171 (76.0) 56 (24.9) 14 (6.2) 11 (4.9)	196 (71.5) 74 (27.0) 17 (6.2) 17 (6.2)
Completed Postgraduate Training, n (%)			
Residency/Fellowship Master’s Degree PhD	14 (28.6) 12 (24.5) 0 (0.0)	102 (45.3) 45 (20.0) 6 (2.7)	116 (42.3) 57 (20.8) 6 (2.2)
Current Job Role			
Not in Clinical Practice ≤25% Clinical Practice 50% Clinical Practice 75% Clinical Practice 100% Clinical Practice	12 (24.5) 2 (4.1) 5 (10.2) 10 (20.4) 20 (40.8)	32 (14.2) 3 (1.3) 12 (5.3) 43 (19.1) 135 (60.0)	44 (16.1) 5 (1.6) 17 (6.2) 53 (19.3) 155 (56.6)

Note: *Not Married includes respondents who were single, separated, divorced, or widowed. **Nuclear family indicates the respondent, their spouse, and any children biological/adopted). ***Other types of training include non-degree diploma courses or specialised clinical courses.

**Figure 2.**
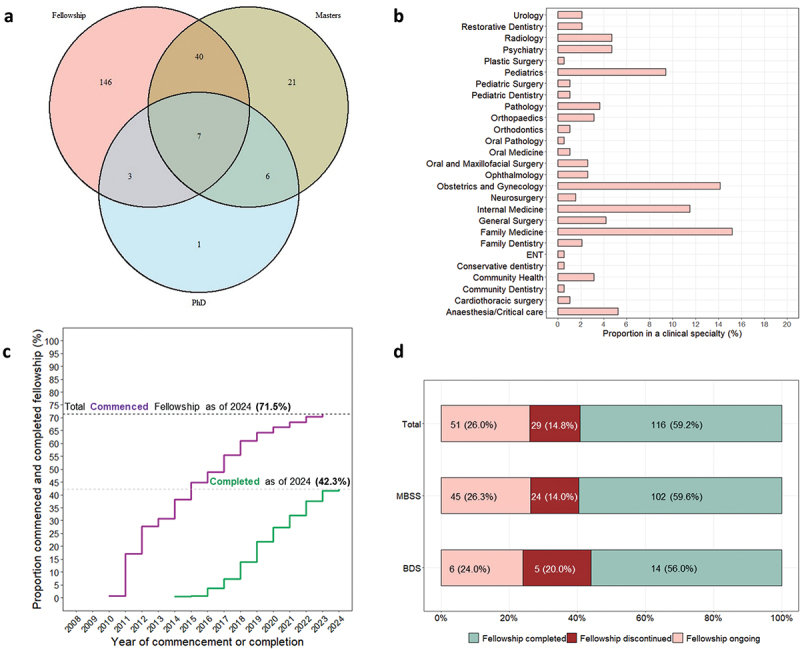
Characteristics of the cohort as of March 2024: (a) number who commenced various postgraduate training; (b) proportion in each clinical specialty (fellowship); (c) proportion who commenced and completed specialty training each year; and (d) overall progress of specialty training.

As of the end of 2023, 42.3% (116/274) of the cohort had qualified as specialists, 20.8% (57/274) had completed a master’s degree, and 2.2% (6/274) had earned a PhD (Table 1). The most common specialties chosen by those who pursued clinical specialist training were Family Medicine (15.8%, 31/196), Obstetrics and Gynaecology (14.3%, 28/196), Internal Medicine (11.2%, 22/196), and Paediatrics (9.2%, 18/196) (Figure 2). It is worth noting that 14.8% (29/196) of those who started training discontinued it, with a higher proportion among dentists (20%) compared to doctors (14%) (Figure 2).

### Prevalence of migration

**Figure 3.**
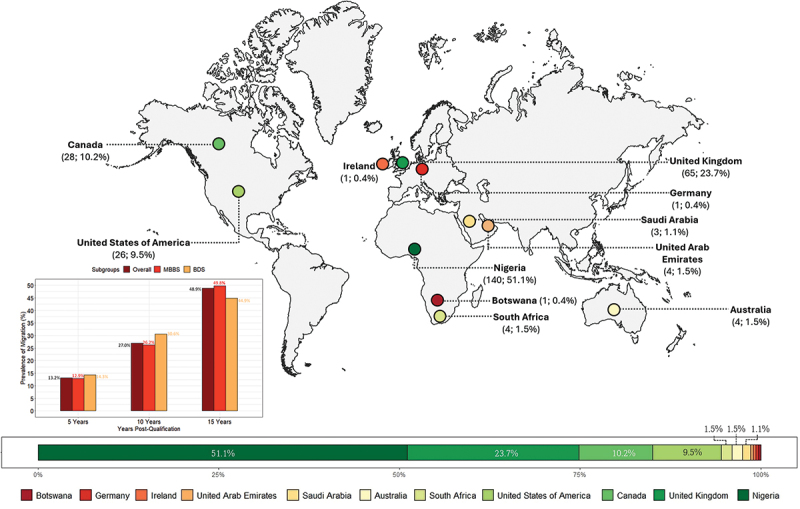
The current location of the 2008 University of Benin graduating medical and dental doctors as of March 2024, and the prevalence of migration at 5-, 10-, and 15-year post-qualification of the cohort (i.e. the figure insert).

The overall prevalence of migration within the cohort doubled approximately every 5 years, rising from 13.2% (36/274) at 5 years post-qualification, to 27.0% (74/274) at 10 years, and reaching 48.9% (134/274) by 15 years. Migration rates among doctors and dentists followed a similar pattern to that of the overall cohort (Figure 3). As of March 2024, only half of the cohort (51.1%, 140/274) remained in Nigeria (Figure 3). Among those who migrated, the vast majority (96.3%, 129/134) relocated outside the African continent. The top three destination countries were the United Kingdom (48.5%, 65/134), Canada (20.9%, 28/134), and the United States of America (19.4%, 26/134).

### Migration incidence rate per person-year

**Figure 4.**
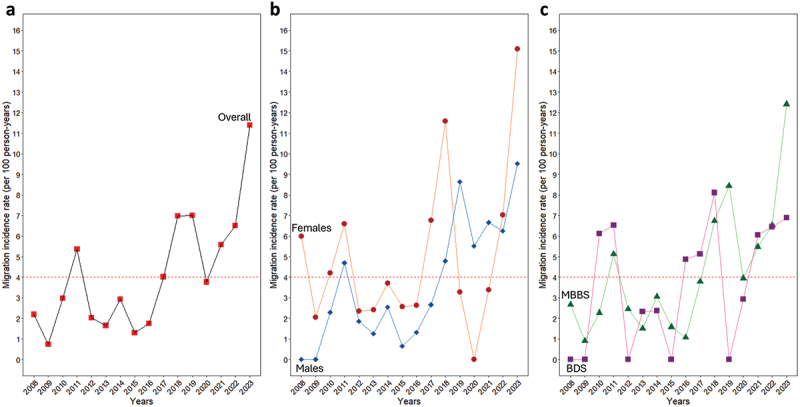
Migration incidence rate per 100 person-years among the 2008 University of Benin graduating medical and dental doctors, 2008 to 2023: (a) overall; (b) by gender; and (c) comparison between medical and dental doctors.

Overall, the cumulative migration incidence rate between 2008 and 2024 was 4 migrations per 100 person-years, with the lowest incidence rate in 2009 (0.74 migrations per 100 person-years) and the highest in 2023 (11.4 migrations per 100 person-years). In the first 8 years (2008–2015) after qualification as doctors and dentists, the incidence rate fluctuated yearly below the cumulative incidence rate, except in 2011, when it was higher. However, from 2016, the yearly incidence rate rose consistently, reaching a plateau in 2018 and 2019 at 7 migrations per 100 person-years (Figure 4a).

An analysis by gender revealed that the yearly migration incidence rate was generally higher among females compared to males until 2018 (Figure 4b). The migration patterns between dentists and doctors were generally similar, except for 2023, where the rate among doctors was nearly double that of dentists (12.4 vs. 6.9 migrations per 100 person-years) (Figure 4c).

### Sociodemographic characteristics associated with migration

**Figure 5.**
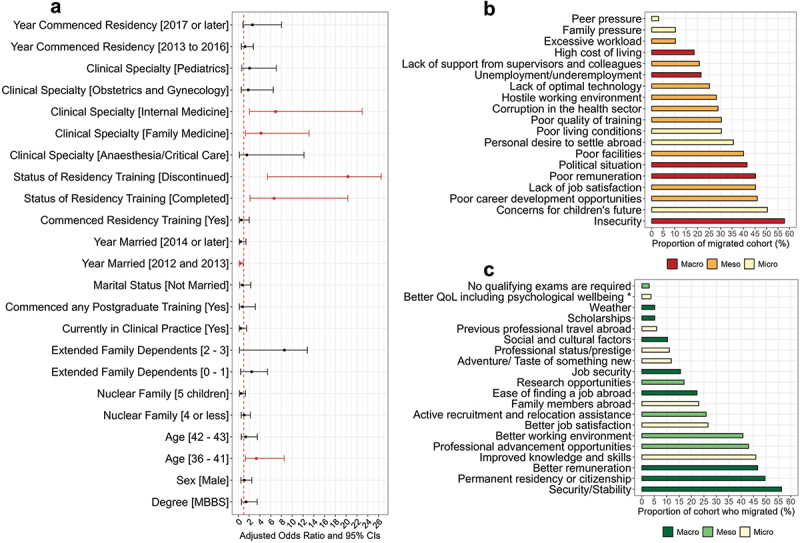
(a) Adjusted odds ratio and 95% confidence interval of individual factors associated with migration; (b) Push factors that led doctors to migrate from Nigeria; and (c) Pull factors that attracted doctors to migrate to destination countries. Note: the vertical dashed red lines indicate the odds ratio of 1. QoL = quality of life.

The reference categories for the logistic regression are shown in the supplementary appendix (Table S1). Age was associated with the decision to migrate, with younger participants (aged 38–41) being three times more likely to migrate compared to their older counterparts (44 years and above) (aOR = 3.3, 95% CI = 1.3–8.5) (Figure 5a). Marriage timing was also associated with migration. Those who got married between 2012 and 2013 were 64% less likely to migrate compared to those who got married in 2011 or earlier (aOR = 0.36, 95% CI = 0.15–0.88). The status of clinical specialist training was further associated with migration. Compared to participants still undergoing training, both those who completed their training (aOR = 6.6, 95% CI = 2.2–20.3) and even those who discontinued training (aOR = 20.3, CI = 5.4–26.5) were significantly more likely to have migrated. Physicians specialising in Family Medicine and Internal Medicine were significantly more likely to migrate, with an aOR of 4.2 (95% CI = 1.3–13.1) and 6.9 (95% CI = 2.1–22.9), respectively.

### Push and pull drivers of migration

The commonest push factors were insecurity of lives and properties (57.8%), followed by concerns about their children’s future (50.3%), limited career development opportunities in Nigeria (45.9%), lack of job satisfaction (45.2%), and poor remuneration (45.2%) (Figure 5b). Among the top five push factors, two (40%) were macro-level factors (insecurity and poor remuneration), two (40%) were meso-level factors (poor career development opportunities and lack of job satisfaction), and one (20%) was a micro-level factor (concerns for children’s future).

The commonest pull factor was the perceived security of lives and properties abroad (56.3%). This was followed by the desire for permanent residency abroad (49.6%), better remuneration (46.7%), opportunities to improve personal knowledge and clinical skills (45.9%), and professional advancement opportunities (42.8%) (Figure 5c). Among the top five pull factors, three (60%) were macro-level factors (security, permanent residency status, and better remuneration), while one (20%) was a meso-level factor (professional advancement opportunities) and one (20%) was a micro-level factor (improved personal knowledge and skills).

## Discussion

In the context of critical shortages of healthcare workers in Nigeria, with numbers far below global targets and further worsened by a continuous exodus of doctors and dentists over the past decade, it is essential to gain a deeper understanding of the patterns and key drivers of migration. This understanding is necessary to guide the development of effective strategies to address the worsening shortages. This retrospective cohort study generated evidence on the temporal patterns and determinants of migration of doctors and dentists from Nigeria. To our knowledge, this is the first cohort study on this topic conducted in sub-Saharan Africa, and it is also the first to examine the temporal pattern of actual migration in the same cohort rather than migration intentions.

We found that the prevalence of migration doubled every 5 years, and as of 15 years post-qualification, about half of this cohort had left Nigeria. More concerning is that among those who have migrated, 9 out of 10 now live and work outside the African continent. Additionally, while the yearly incidence rate of migration remained stable during the first 8 years after graduation, it has spiked since 2016. Similar to other studies conducted in Nigeria [12,14], South Africa [28], and a systematic review of studies from over 90 LMICs [13], our research also found that the top three destination countries for emigrating doctors and dentists are the UK, the USA, and Canada. Insecurity, concerns for children’s future, poor career development opportunities, lack of job satisfaction, and inadequate remuneration were the commonest factors driving the migration of this cohort from Nigeria, similar to findings from elsewhere in sub-Saharan Africa [28–30].

The finding that nearly half of this cohort has emigrated 15 years after qualifying, with the prevalence doubling every 5 years, underscores the dire consequences for Nigeria’s healthcare system. The country’s already low doctor-to-population ratio of 3.9 per 10,000 falls far short of recommended standards [11], exacerbating health disparities and hindering progress towards the Sustainable Development Goals. Despite having a relatively large capacity for training the healthcare workforce, Nigeria faces a per capita deficit due to the absence of strategic policies for training, placement, and retention [31,32]. If the current migration trend continues, critical doctor and dentist shortages will worsen, requiring years and substantial efforts just to maintain the existing doctor-to-population ratio. The fact that more than 95% of those who emigrated now live and work outside the African continent is particularly concerning. Africa, with 13.76% of the global population and 25% of the global disease burden, has only 1.3% of the global healthcare workforce [33]. At the turn of the millennium, Africa’s doctor-to-population ratio had declined to 0.2 doctors per 10,000, compared to 24 per 10,000 in the USA and 28 per 10,000 in the UK [9]. With a relatively large absolute number of doctors and dentists, the continent could have benefited from Nigeria’s loss if these migrations were to other African countries.

The stable incidence rate of migration during the initial 8 years after graduation for this cohort is not surprising. Several policies, economic, and professional events before and during this period may explain the observed patterns. Barely a year after this cohort graduated, the Federal Government of Nigeria approved the Consolidated Medical Salary Scale (CONMESS) in September 2009 [34]. CONMESS marked a watershed moment for Nigerian doctors and dentists, substantially increasing their compensation packages compared to previous pay structures. Additionally, the country experienced consistent positive growth in Gross Domestic Product (GDP) per capita in the decade leading up to 2008 [35], which persisted until 2016, when the country entered a recession [36]. The increase in compensation package due to CONMESS and the economic stability offered renewed hope at a time when this cohort was starting their careers, potentially contributing to the relatively stable migration rate in the initial years. Furthermore, this period coincided with the start of postgraduate specialist training (residency) for most cohort members, likely committing them to Nigeria until they completed their qualifications. Taken together, the stable yearly incidence rate of migration observed early in the careers of this cohort underscores the significant impact that government policies on compensation, economic stability, and clear professional paths can have on health workforce retention.

The rising yearly incidence rate of migration, since 2016, may be attributed to several factors, including government policy actions, Nigeria’s economic situation during the study period, and the professional stage of the cohort. Although the introduction of CONMESS had a net positive effect on doctors and dentists’ compensation, its implementation was inconsistent, with many sub-national governments delaying its adoption [37]. This inconsistency led to several nationwide industrial actions. In 2014, 2 years prior to the rise in migration, the Federal Government of Nigeria sacked all 16,000 resident doctors and dentists following a month-long industrial action [38]. Although this was eventually resolved through negotiations, another doctors and dentists’ strike occurred in 2016 due to demands for an upward review of compensation. Again, the government responded by sacking all striking resident doctors and suspending the Residency Training Programme indefinitely [39]. Notably, from 2016 onward, the first set of cohort members completed their residency and qualified as specialists. This coincided with an economic recession and difficulty in securing consultant-level appointments, leading many newly qualified specialists to be under-employed. During this period, aggressive recruitment efforts by foreign countries offering more attractive compensation packages were also prevalent [40,41]. These combined factors likely created an unstable work environment and a sense of precarity, which may partly explain the escalating migration pattern from 2016 onward.

Worsening insecurity, concerns for children’s future, poor career development opportunities, lack of job satisfaction, and inadequate remuneration were the most common push factors. Several other studies from Nigeria have reported similar factors [12,14,15], although they have primarily focused on the ‘intention to migrate’ rather than actual migration. Nigeria is reported as one of the most insecure countries in Africa. For example, in 2022, the Armed Conflict Location & Event Data Project (ACLED) estimated approximately 3,700 political violence events and 3,900 fatalities resulting from violence targeting civilians [42]. The worsening insecurity is attributable to many factors, including the Boko Haram insurgency in northeast Nigeria, banditry and kidnapping in southern Nigeria, clashes between herders and farmers in central Nigeria, conflicts between Nigeria’s security forces and separatist insurgents in southeast Nigeria, and militancy in the oil-rich Niger Delta region [42–44]. In this state of general insecurity, healthcare workers, their relatives, and families are not spared. For example, there have been repeated reports of doctors and dentists being targeted, kidnapped, or attacked during the course of their routine duties [45,46]. Both the national and subnational governments of Nigeria must make concerted efforts to understand and address the root causes of this complex problem. This will not only help retain doctors and dentists in Nigeria but also attract those from abroad to return, potentially improving the worsening doctor-to-population ratio in the country.

Our findings have important implications and provide granular evidence to guide doctor and dentist retention strategies in Nigeria and other LMICs facing similar healthcare workforce migration issues. Nigeria’s healthcare system stands at a crucial turning point. The country’s population is projected to double from around 200 million in 2019 to an estimated 400 million by 2050 and 733 million by 2100 [47], positioning it as the world’s third most populous country. Furthermore, Nigeria continues to struggle with poor health outcomes, showing insufficient progress over the past three decades. Health investment has consistently fallen below recommended global and regional targets, and the limited resources allocated have been mismanaged by successive governments since gaining independence [31]. To prevent a looming crisis, the government must prioritise tackling the root causes of the rising emigration of its health workforce. This includes addressing macro and meso-level factors such as insecurity, inadequate remuneration, limited career development opportunities, and a lack of job satisfaction as identified in this study. These factors are intertwined with the broader socioeconomic and political challenges. Addressing these broader macro and meso-level issues could potentially have a ripple effect in also alleviating the micro-level factors of migration such as concerns about children’s future.

In response to the massive exodus of doctors and dentists, the Nigerian legislature introduced the Medical and Dental Practitioners Act (Amendment) Bill in 2022 [48]. This bill sought to mandate a five-year post-qualification work period for doctors and dentists trained in Nigeria. However, it presented an oversimplified solution to a complex and multifaceted issue, failing to address the root causes of migration and lacking evidence-based justification [49]. In August 2024, the Nigerian government introduced the National Policy on Health Workforce Migration [50], which focuses on retaining healthcare workers through incentives, particularly for those serving in rural and underserved areas, and encouraging diaspora return. Key incentives for health workers include regular reviews of remuneration, special mortgage schemes, health insurance, tax breaks, and improved working conditions. Additionally, the policy promotes career support, training opportunities, expansion of training institutions, data-driven approaches to monitoring of health worker migration, and bilateral agreements with destination countries [50].

While the policy is a step in the right direction and addresses many of the macro and meso-level drivers of migration identified in this study, there are potential bottlenecks to its success. First, the lack of specificity regarding the review of remuneration may hinder its effectiveness [51]. Second, the potential for uneven implementation by sub-national governments, as seen with the inconsistent rollout of CONMESS in 2009, could undermine the effectiveness of this policy. Third, the policy’s funding source remains unclear. Inadequate funding is a common cause of policy failure in LMICs, and Nigeria has yet to meet its 2001 Abuja Declaration commitment to allocate at least 15% of annual expenditure to health [52]. In 2024, only 4.6% was allocated, down from a high of 5.7% in 2023 [53], raising serious concerns about the feasibility of securing the necessary resources. Lastly, insecurity of lives and property – the leading macro-level push factor of migration in this study – cannot be addressed by this policy, as it falls outside the purview of the Ministry of Health. Tackling this issue requires a more comprehensive approach and strong political will from both the sub-national and central governments.

Our study has some limitations that warrant cautious interpretation of the findings. First, focusing on a single Nigerian medical school may limit the generalisability of the findings to the broader national context, as regional variations in migration patterns are possible. Additionally, more recent graduating cohorts may exhibit different migration trends. Thus, further research should involve medical and dental school cohorts from the different geopolitical regions of Nigeria and include more recently graduated cohorts to explore potential variations in migration patterns. Second, members of the research team who were also part of the 2008 UNIBEN Medical School cohort were included as respondents. While this could introduce potential bias, the team’s composition closely mirrored that of the cohort, with 52% (12/23) living and working in Nigeria, similar to 51.1% (140/274) of the entire cohort. Therefore, their inclusion is unlikely to have biased the findings. Third, despite extensive efforts, some cohort members were untraceable, and some participants did not complete the questionnaire. These individuals may differ on unmeasurable characteristics, but the high response rate of over 72% supports the validity and reliability of our results.

Fourth, we only captured information about the participants’ current country of residence, without considering their migratory pathways or movements between multiple countries. This limits our ability to analyse the full migration trajectory and understand the mobility dynamics of the cohort. Future studies on this cohort could explore migratory pathways to provide a more nuanced understanding of migration patterns. Fifth, understanding the broader continuum of migration is essential for effective policy guidance. In a forthcoming mixed-methods study, we will explore additional dimensions, such as ‘intention to migrate’ and ‘return migration’. Finally, retrospective cohort studies have inherent limitations. While we discuss associations between observed migration patterns and various social, political, economic, and personal factors in Nigeria during the study period, establishing definitive cause-and-effect relationships is challenging due to potential unmeasured confounders. Despite these limitations, our findings offer valuable insights into how many members of the cohort have migrated, their destinations, and the factors influencing their migration from Nigeria.

## Conclusion

Our study highlights the critical issue of doctor and dentist migration from Nigeria, emphasising the alarming trends and underlying factors driving this phenomenon. The significant exodus of nearly half of the 2008 cohort within 15 years of graduation highlights the urgent need for strategic interventions to address the root causes of this migration. Insecurity, inadequate remuneration and concerns about career prospects and children’s futures were the key push factors driving doctors and dentists to seek better opportunities abroad. The sharp increase in migration rates post-2016 underscores the impact of inconsistent government policies, economic instability, and professional challenges faced by healthcare workers in Nigeria.

Our findings suggest that unless comprehensive and effective measures are taken, Nigeria’s healthcare system will face severe shortages of skilled professionals, exacerbating the already poor doctor-to-population ratio and impeding progress towards achieving equitable healthcare. To avert this looming crisis, the Nigerian government must prioritise addressing the intertwined issues of security, economic stability, and professional development. Strategic healthcare investments, coupled with effective governance and anti-corruption measures, are essential to retain healthcare workers and ensure the sustainability of Nigeria’s healthcare system.

## Supplementary Material

Supplementary appendix.docx

## Data Availability

The dataset generated and analysed during the current study is not publicly available due to the need to protect participant confidentiality but is available upon reasonable request from the corresponding author. All the R codes developed for the purposes of the analysis reported here are freely available in GitHub, an open repository at: https://github.com/drwariri/Migration-patterns-and-determinants-15-year-retrospective-cohort-study.

## Notes

The study received ethical approval (CMS/REC/2023/457; Date: 23 October 2023) from the Research Ethics Committee, College of Medical Sciences, University of Benin, Edo state, Nigeria. All study participants provided informed consent to participate.
