# Supplementary material for: Understanding the exodus: a 15-year retrospective cohort study on the pattern and determinants of migration among Nigerian doctors and dentists
Source: Glob Health Action. 2024 Nov 29;17(1):2432754. doi: 10.1080/16549716.2024.2432754 (PMC11610232; doi:10.1080/16549716.2024.2432754)
Supplement: Supplementary appendix.docx [file ZGHA_A_2432754_SM0034.docx]

**Supplementary material**

**Understanding the Exodus: A 15-Year Retrospective Cohort Study on the Pattern and Determinants of Migration Among Nigerian Medical Doctors and Dentists**

# Reference categories of all the covariates in the regression analysis

**Table S1**: The reference categories of all the sociodemographic characteristics associated with migration included in the bivariate and multivariate regression analysis

|  | **Covariate (variable)** | **Reference category** |
| --- | --- | --- |
| 1. | Degree | BDS |
| 2. | Sex | Female |
| 3. | Age | 44 – 46 years (i.e., above mean age) |
| 4. | Nuclear Family Size | 6 or more |
| 5. | Extended Family Size | 4 or more dependents |
| 6. | Currently in Clinical Practice ? | No |
| 7. | Commenced any Postgraduate Training? | No |
| 8. | Marital Status | Married |
| 9. | Year Married | 2011 or earlier |
| 10. | Commenced Residency Training? | No |
| 11. | Status of Residency Training | Currently in training |
| 12. | Clinical Specialty | Others* |
| 13. | Year Commenced Residency | 2010 to 2012 |

* Note: "Others" includes all specialties apart from Pediatrics, Obstetrics and Gynecology, Internal Medicine, Family Medicine, and Anaesthesia/Critical Care. These specialties each represented less than 5% of the cohort who had commenced residency training and were thus aggregated into a single category.

# Unadjusted bivariate analysis of associated factors


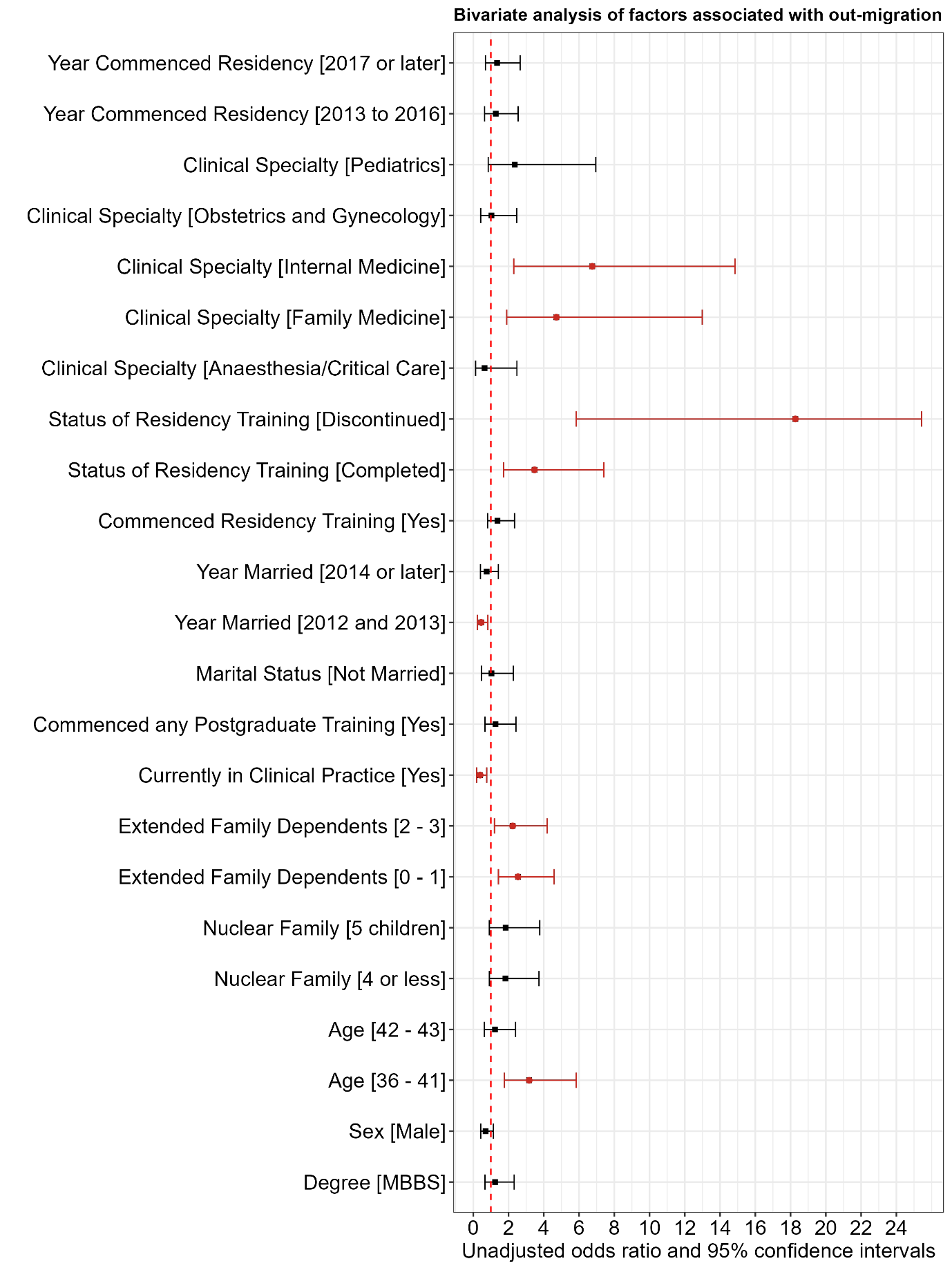


**Figure S1**: Unadjusted odds ratio and corresponding 95% credible interval plots for the sociodemographic determinants of migration. **Note:** The vertical dashed red lines mark the Odds Ratio of 1. Red dots and lines show the unadjusted odds ratios and 95CIs of variables with significant associations with out-migration among the cohort.

# Study questionnaire

Study Number: |_|_|_|_|_|_|_|_|_|_|

Section 1- Demographic Information

- 1. Age (in years) as at the last birthday?

Ans: drop down with numbers 35 to 65.

- 1. What is your gender?

(Multiple choice, one response allowed)

- Male
- Female
  1. What is your marital status?

(Multiple choice, one response allowed)

- Single
- Married
- Separated
- Divorced
- Widowed
  1. What year did you get married

Ans: drop-down choice with years

- 1. What is the size of your nuclear family?

Ans: drop-down choice with numbers 1 to 15.

- 1. How many other dependents do you have, not counting spouses or children?

Ans: drop-down choice with numbers 0 to 25.

Section 2- Career Paths

- 1. What degree was awarded to you upon graduation from the University of Benin in 2008?

(Multiple choice, one response allowed)

- Bachelor of Dental Surgery (BDS)
- Bachelor of Medicine, Bachelor of Surgery (MBBS)
  1. Did you commence any postgraduate training?

(Multiple choice, one response allowed)

- Yes

- No (If no, skip to question 2.4)

- 1. What type of postgraduate training did you undertake? Tick all that apply.

(Multiple choice, multiple responses allowed)

- Residency/Fellowship (Will answer questions 2.3.1.1 to 2.3.1.)

- Masters

- PhD

- Other (Free text option)

- - 1. If you ticked Residency/Fellowship, please answer the following questions.
       1. What residency/fellowship training did you undertake?

Ans: Free Text

- - - 1. How many years post-MBBS/BDS did training commence?

Ans: drop-down choice with numbers 0 to 15.

- - - 1. Regarding residency training- Please pick the option that applies to you, multiple choice with branching questions)

- - 1. If you ticked Masters, please answer the following questions.
       1. What master’s degree(s) did you undertake? Option to add more than one response.

Ans: free text

+ Add another.

- - - 1. How many years post-MBBS/BDS did training commence?

Ans: drop-down choice with numbers 0 to 15.

- - - 1. For master’s degree training- Please pick the option that applies to you, multiple choice with branching questions)
    1. If you ticked Ph.D., please answer the following questions.
       1. What Ph.D. degree did you undertake? Option to add more than one response.

Ans: free text

+ Add another.

- - - 1. How many years post-MBBS/BDS did training commence?

Ans: drop-down choice with numbers 0 to 15.

- - - 1. For PhD degree training- Please pick the option that applies to you, multiple choice with branching questions)
    1. If you ticked other training, please answer the following questions.

2.3.5.1 What other postgraduate degree did you undertake?

Ans: free text

+ Add another.

- - - 1. How many years post-MBBS/BDS did training commence?

Ans: drop-down choice with numbers 0 to 15.

- - - 1. For other postgraduate degree training- Please pick the option that applies to you, multiple choice with branching questions)
  1. Are you in clinical practice, i.e., do you see actively patients?

- Yes

- No (If no, skip to question 2.5)

- - 1. If you answered yes to Question 2.4, on average, what percentage of your job is clinical practice? (Multiple choice, single response allowed)
- Less than 25%
- About 50%
- About 75%
- Practically 100%
  1. What is your current **PRIMARY** role?

Ans: free text option

- 1. How many years have you been in your current role?

Ans: drop-down choice with numbers 0 to 15.

- 1. Do you have other secondary roles? If so, please indicate (allow multiple responses)

- I do not have any other roles

- Teaching

- Research

- Administrative

- Other (free text option)

- 1. What **best** describes your **PRIMARY** job sector? (Multiple choice, single response allowed)
- Government
- Research or teaching institutions
- Private Office
- Non-Governmental Organizations (NGO)

Section 3- Current location

- 1. Do you currently live in Nigeria?

(Multiple choice, one response allowed)

- Yes (If yes, go to question 3.3)

- No

- 1. What country do you live in? (Single response allowed)

Ans: drop-down choice with list of countries

- - 1. What year did you leave Nigeria?

Ans: drop-down option with choice of years 2008 to 2023 (Skip to Section 4)

- 1. Do you intend to leave Nigeria in the future?

- Yes

- No (If no, skip to section 5)

- - 1. What is your likely destination country? Pick up to 3 if applicable.

Ans: drop-down choice with list of countries

Section 4: Drivers of out-migration/intention-to-migrate.

4.1. What factors in Nigeria influenced your decision to leave Nigeria or intention to leave to work in another country? (Pick the factors that apply to you)

- Unemployment/underemployment

- Insecurity

- Political situations in the country

- poor living conditions

- High cost of living

- Lack of job satisfaction

- Poor career development opportunities/prospects

- Poor quality of training

- Corruption in the health sector including nepotism

- Poor remuneration

- Hostile working environment

- Excessive workload

- Lack of professional support from supervisors/colleagues

- Poor facilities

- lack of optimal technology

- Peer pressure

- Family pressure

- personal ambition/desire to settle abroad

- concerns for children's future

**Others:** please list (free text)

4.2 What factors influenced the choice of the destination country to which you migrated or intend to migrate to?  (Pick the factors that apply to you)

- Security/Stability

-Conducive immigration and settlement policies in the host country including offer of permanent residency or citizenship

- Active recruitment of health workers’ relocation assistance

- Better remuneration

- Scholarships

- Better working environment

- Professional advancement opportunities

- Better quality of training/Improved knowledge and skills

- research opportunities

- Easy of finding a job abroad

- Better job satisfaction

- Professional status/prestige

- Job security

- No qualifying exams are required to practice

- Better quality of life including psychological wellbeing

- Peer Influence

- Adventure/ Taste of something new

- Family members abroad

- weather

- Previous travel abroad for professional reasons

- Social and cultural factors including language, cultural similarities, or personal freedom

**Others:** please list (free text)

4.3 Did you consider other countries for migration?

(Multiple choice, one response allowed)

- Yes

- No (If no, go to section 6)

4.3.1 Which other countries did you consider for migration? Pick up to 3 if applicable.

Ans: drop-down choice with list of countries

4.3.2 What factors discourage(d) you from migrating to those countries? (Pick the factors that apply to you)

- racism

- language barrier

- religion and culture

- qualifying examinations

- poor career prospects including a lack of information about career pathway

- poor working conditions including long working hours

- Inadequate remuneration

- lack of professionalism and respect

- cost of migration/ financial barriers

- weather conditions

- Poor quality of life including fractured family life

- Lack of opportunities for family members including lack of job opportunities for spouse or educational opportunities for children

**Others:** please list (free text)

4.4 What factors will influence your decision to return to Nigeria?

Ans [Free text]

Section 5: Drivers of non-migration

What factors influenced your decision not to migrate out of Nigeria? (Survey end)

- Bonds
- Racism in other countries
- language barrier in other countries
- religion and culture of other countries
- Good remuneration in Nigeria
- Professional satisfaction including professional accomplishment in Nigeria.
- Opportunity for career advancement in Nigeria
- Job security in Nigeria
- Good working conditions in Nigeria
- qualifying examinations to practice in other countries
- poor career prospects including a lack of information about career pathway in other countries
- Poor working conditions including long working hours in other countries
- Inadequate remuneration in other countries
- Family ties in Nigeria
- Advancing age
- Culture and social life in Nigeria
- Desire to raise children in Nigeria.
- Religion in Nigeria
- Lack of necessary resources to migrate out of Nigeria.
- Having financial security including ownership of business or properties in Nigeria
- Patriotism including the desire to settle in Nigeria/optimism about the future in Nigeria.
- weather conditions in other countries
- Poor quality of life including fractured family life in other countries
- Lack of opportunities for family members in other countries including lack of job opportunities for spouses or educational opportunities for children

**Others: please list (free text)**

Section 6: Drivers of return migration

- 1. Did you ever migrate from Nigeria and **then RETURN**?

- Yes

- No (If no, skip to end of survey)

- 1. What year did you leave? (Single response allowed)
- drop-down option with choice of N/A, and years 2008 to 2023
  1. Which country did you go to?

Ans: drop-down choice with list of countries

- 1. What year did you return? (Single response allowed)

drop-down option with choice of N/A, and years 2008 to 2023

- 1. If you migrated out of Nigeria **AND RETURNED**, what factors influenced your decision to leave your destination country and return to Nigeria?

Ans [Free text]

Section 7: End of survey for all respondents

7.1 Would you like to be contacted for the focus group discussion on this topic?

- Yes

- No

# Description of the variables in the dataset

|  | **Variable** | **Description of variable** |
| --- | --- | --- |
| 1 | age | Age as of last birthday at completion of questionnaire. |
| 2 | sex | Sex of participant, either male or female. |
| 3 | married | Indicate marital status of the participants (married, single, divorced or separated). |
| 4 | yr_married | Year participants got married. |
| 5 | yr_divorced | Year participants got divorced. |
| 6 | fm_size | Indicate size of nuclear family (i.e., spouse and children). |
| 7 | dependents | Number of extended family dependent on study participant. |
| 8 | degree | Degree awarded at graduation in 2008 (BDS or MBBS). |
| 9 | commence_training | Indicate if they commenced postgraduate training (residency, masters, or PhD). Option is yes or no. |
| 10 | training_type | Indicate the combination of postgraduate training participant commenced. Options include residency/masters/PhD, etc. |
| 11 | fellowship | Indicate if they commenced residency/fellowship. Option are 0 = no, and 1 = yes. |
| 12 | masters | Indicate if they commenced masters. Option are 0 = no, and 1 = yes. |
| 13 | PhD | Indicate if they commenced PhD. Option are 0 = no, and 1 = yes. |
| 14 | training_other | Indicate if they commenced any other training apert from residency, masters and PhD. Option are 0 = no, and 1 = yes. |
| 15 | fellow_type | Indicate the sub-specialty or field of residency training. Specific areas of training or sub-specialties are listed. |
| 16 | fellow_yr | Indicate which year participant commenced fellowship/residency training. |
| 17 | fellow_status | Indicates if the participant completed, discontinued or if their fellowship training is currently ongoing. |
| 18 | fellow_comp_yr | Indicate what year participant completed their fellowship training. |
| 19 | fellow_country | Indicate which country the study participant did their fellowship/residency training. |
| 20 | fellow_expected_yr | Indicate what year those who are currently undergoing their fellowship/residency training expect to complete their training. |
| 21 | fellow_discont_yr | Indicate what year participant discontinued their fellowship or residency training. |
| 22 | masters_type | Indicate the type of masters degree participant commenced. Specific areas of training or sub-specialties are listed. |
| 23 | masters_yr | Indicate which year participant commenced masters. |
| 24 | masters_status | Indicates if the participant completed, discontinued or if their masters training is currently ongoing. |
| 24 | masters_comp_yr | Indicate what year participant completed their masters degree. |
| 26 | masters_country | Indicate which country the study participant did their masters. |
| 27 | masters_expected_yr | Indicate what year those who are currently undergoing their masters degree expect to complete their training. |
| 28 | masters_discont_yr | Indicate what year participant discontinued their masters degree training. |
| 29 | PhD_type | Indicate the field of PhD training. |
| 30 | PhD_yr | Indicate which year participant commenced PhD training. |
| 31 | PhD_status | Indicates if the participant completed, discontinued or if their PhD training is currently ongoing. |
| 32 | PhD_comp_yr | Indicate what year participant completed their PhD training. |
| 33 | PhD_country | Indicate which country the study participant did their PhD training. |
| 34 | clinical_pract | Indicate if the study participant is in clinical practice or if they actively see patients. The answers are yes or no |
| 35 | percent_clin_pract | Estimated proportion of time the study participant devote to clinical practice. |
| 36 | pri_job_descrip | Indicates the study participant’s primary job role or their job description. |
| 37 | yrs_pri_role | Indicates how many years the study participant have been on their current primary job role |
| 38 | sec_job_descrip | Indicates the study participant’s other job roles or description (i.e., secondary job roles). |
| 39 | sec_job_no | Indicates if they do not have other secondary job roles. 1 = they do not have other roles, 0 = indicates they have other roles. |
| 40 | sec_job_teach | Indicates if teaching is their other secondary job roles. 1 = teaching is a secondary role, 0 = teaching not secondary role. |
| 41 | sec_job_research | Indicates if research is their other secondary job roles. 1 = research is a secondary role, 0 = research not secondary role. |
| 42 | sec_job_admin | Indicates if admin is their other secondary job roles. 1 = admin is a secondary role, 0 = admin not secondary role. |
| 43 | pri_job_sector | Indicates what sector the study participant’s primary job role is in. |
| 44 | pri_job_govt | Indicates government sector. 1 = government sector, 0 = not government sector. |
| 45 | pri_job_research | Indicates research/teaching sector. 1 = research/teaching sector, 0 = not research/teaching sector. |
| 46 | pri_job_private | Indicates private sector. 1 = private sector, 0 = not private sector. |
| 47 | pri_job_ngo | Indicates NGO sector. 1 = private sector, 0 = not NGO sector. |
| 48 | pri_job_other | Indicates other sector. 1 = private sector, 0 = not other sector. |
| 49 | migrate | Indicate if the study participant is still in Nigeria. Yes = not migrated, No = migrated from Nigeria. |
| 50 | migrate_country | Indicate which country the study participant have migrated to |
| 51 | migrate_yr | Indicate which year the study participant migrated from Nigeria |
| 52 | intention_migrate | Intention to migrate for those still in Nigeria. Options: yes or no |
| 53 | intention_country | Indicate which country the study participant intend to migrate to |
| 54 | migrate_drivers | Indicate what factors determined migration among participants who have migrated (**push factors**). Multiple response. |
| 55 | migrate_drivers_1 | Indicate if **Unemployment/underemployment** determined their migration. Yes = 1, No = 0. |
| 56 | migrate_drivers_2 | Indicate if **Insecurity** determined their migration. Yes = 1, No = 0. |
| 57 | migrate_drivers_3 | Indicate if **Political situation in the country** determined their migration. Yes = 1, No = 0. |
| 58 | migrate_drivers_4 | Indicate if **poor living conditions** determined their migration. Yes = 1, No = 0. |
| 59 | migrate_drivers_5 | Indicate if **High cost of living** determined their migration. Yes = 1, No = 0. |
| 60 | migrate_drivers_6 | Indicate if **Lack of job satisfaction** determined their migration. Yes = 1, No = 0. |
| 61 | migrate_drivers_7 | Indicate if **Poor career development opportunities/prospects** determined their migration. Yes = 1, No = 0. |
| 62 | migrate_drivers_8 | Indicate if **Poor quality of training** determined their migration. Yes = 1, No = 0. |
| 63 | migrate_drivers_9 | Indicate if **Corruption in the health sector including nepotism** determined their migration. Yes = 1, No = 0. |
| 64 | migrate_drivers_10 | Indicate if **Poor remuneration** determined their migration. Yes = 1, No = 0. |
| 65 | migrate_drivers_11 | Indicate if **Hostile working environment** determined their migration. Yes = 1, No = 0. |
| 66 | migrate_drivers_12 | Indicate if **Excessive workload** determined their migration. Yes = 1, No = 0. |
| 67 | migrate_drivers_13 | Indicate if **Lack of professional support from supervisors and colleagues** determined their migration. Yes = 1, No = 0. |
| 68 | migrate_drivers_14 | Indicate if **Poor facilities** determined their migration. Yes = 1, No = 0. |
| 69 | migrate_drivers_15 | Indicate if **lack of optimal technology** determined their migration. Yes = 1, No = 0. |
| 70 | migrate_drivers_16 | Indicate if **Peer pressure** determined their migration. Yes = 1, No = 0. |
| 71 | migrate_drivers_17 | Indicate if **Family pressure** determined their migration. Yes = 1, No = 0. |
| 72 | migrate_drivers_18 | Indicate if **Personal ambition/desire to settle abroad** determined their migration. Yes = 1, No = 0. |
| 73 | migrate_drivers_19 | Indicate if **Concerns for children's future** determined their migration. Yes = 1, No = 0. |
| 74 | intention_drivers | Indicate what factors determine intention to migrate among participants **(push factors)**. Multiple response. |
| 75 | intention_drivers_1 | Indicate if **Unemployment/underemployment** determines their intention to migrate. Yes = 1, No = 0. |
| 76 | intention_drivers_2 | Indicate if **Insecurity** determines their intention to migrate. Yes = 1, No = 0. |
| 77 | intention_drivers_3 | Indicate if **Political situation in the country** determines their intention to migrate. Yes = 1, No = 0. |
| 78 | intention_drivers_4 | Indicate if **poor living conditions** determines their intention to migrate. Yes = 1, No = 0. |
| 79 | intention_drivers_5 | Indicate if **High cost of living** determines their intention to migrate. Yes = 1, No = 0. |
| 80 | intention_drivers_6 | Indicate if **Lack of job satisfaction** determines their intention to migrate. Yes = 1, No = 0. |
| 81 | intention_drivers_7 | Indicate if **Poor career development opportunities/prospects** determines their intention to migrate. Yes = 1, No = 0. |
| 82 | intention_drivers_8 | Indicate if **Poor quality of training** determines their intention to migrate. Yes = 1, No = 0. |
| 83 | intention_drivers_9 | Indicate if **Corruption in the health sector including nepotism** determines their intention to migrate. Yes = 1, No = 0. |
| 84 | intention_drivers_10 | Indicate if **Poor remuneration** determines their intention to migrate. Yes = 1, No = 0. |
| 85 | intention_drivers_11 | Indicate if **Hostile working environment** determines their intention to migrate. Yes = 1, No = 0. |
| 86 | intention_drivers_12 | Indicate if **Excessive workload** determines their intention to migrate. Yes = 1, No = 0. |
| 87 | intention_drivers_13 | Indicate if **Lack of professional support from supervisors and colleagues** determines their intention to migrate. Yes = 1, No = 0. |
| 88 | intention_drivers_14 | Indicate if **Poor facilities** determines their intention to migrate. Yes = 1, No = 0. |
| 89 | intention_drivers_15 | Indicate if **lack of optimal technology** determines their intention to migrate. Yes = 1, No = 0. |
| 90 | intention_drivers_16 | Indicate if **Peer pressure** determines their intention to migrate. Yes = 1, No = 0. |
| 91 | intention_drivers_17 | Indicate if **Family pressure** determines their intention to migrate. Yes = 1, No = 0. |
| 92 | intention_drivers_18 | Indicate if **Personal ambition/desire to settle abroad** determines their intention to migrate. Yes = 1, No = 0. |
| 93 | intention_drivers_19 | Indicate if **Concerns for children's future** determines their intention to migrate. Yes = 1, No = 0. |
| 94 | destination_factors | Factors that determined the choice of country for participants who have migrated **(pull factors)**. Multiple response. |
| 95 | destination_factors_1 | **Security/Stability** determined the country of choice (pull factor). Yes = 1, No = 0. |
| 96 | destination_factors_2 | **Conducive immigration and settlement policies in the host country including offer of permanent residency or citizenship** determined the country of choice (pull factor). Yes = 1, No = 0. |
| 97 | destination_factors_3 | **Active recruitment of health workers including relocation assistance** determined the country of choice (pull factor). Yes = 1, No = 0. |
| 98 | destination_factors_4 | **Better remuneration** determined the country of choice (pull factor). Yes = 1, No = 0. |
| 99 | destination_factors_5 | **Scholarships** determined the country of choice (pull factor). Yes = 1, No = 0. |
| 100 | destination_factors_6 | **Better working environment** determined the country of choice (pull factor). Yes = 1, No = 0. |
| 101 | destination_factors_7 | **Professional advancement opportunities** determined the country of choice (pull factor). Yes = 1, No = 0. |
| 102 | destination_factors_8 | **Improved knowledge and skills** determined the country of choice (pull factor). Yes = 1, No = 0. |
| 103 | destination_factors_9 | **Research opportunities** determined the country of choice (pull factor). Yes = 1, No = 0. |
| 104 | destination_factors_10 | **Ease of finding a job abroad** determined the country of choice (pull factor). Yes = 1, No = 0. |
| 105 | destination_factors_11 | **Better job satisfaction** determined the country of choice (pull factor). Yes = 1, No = 0. |
| 106 | destination_factors_12 | **Professional status/prestige** determined the country of choice (pull factor). Yes = 1, No = 0. |
| 107 | destination_factors_13 | **Job security** determined the country of choice (pull factor). Yes = 1, No = 0. |
| 108 | destination_factors_14 | **No qualifying exams are required to practice** determined the country of choice (pull factor). Yes = 1, No = 0. |
| 109 | destination_factors_15 | **Better quality of life including psychological wellbeing** determined the country of choice (pull factor). Yes = 1, No = 0. |
| 110 | destination_factors_16 | **Adventure/ Taste of something new** determined the country of choice (pull factor). Yes = 1, No = 0. |
| 111 | destination_factors_17 | **Family members abroad** determined the country of choice (pull factor). Yes = 1, No = 0. |
| 112 | destination_factors_18 | **Weather** determined the country of choice (pull factor). Yes = 1, No = 0. |
| 113 | destination_factors_19 | **Previous travel abroad for professional reasons** determined the country of choice (pull factor). Yes = 1, No = 0. |
| 114 | destination_factors_20 | **Social and cultural factors including language, cultural similarities, or personal freedom** determined the country of choice (pull factor). Yes = 1, No = 0. |
| 115 | intent_dest_factors | Factors that determined the choice of country for participants intend to migrate to **(pull factors)**. Multiple response. |
| 116 | intent_dest_factors_1 | **Security/Stability** determines the country of choice participant intend to migrate. Yes = 1, No = 0. |
| 117 | intent_dest_factors_2 | **Conducive immigration and settlement policies in the host country including offer of permanent residency or citizenship** determines the country of choice participant intend to migrate. Yes = 1, No = 0. |
| 118 | intent_dest_factors_3 | **Active recruitment of health workers including relocation assistance** determines the country of choice participant intend to migrate. Yes = 1, No = 0. |
| 119 | intent_dest_factors_4 | **Better remuneration** determines the country of choice participant intend to migrate. Yes = 1, No = 0. |
| 120 | intent_dest_factors_5 | **Scholarships** determines the country of choice participant intend to migrate. Yes = 1, No = 0. |
| 121 | intent_dest_factors_6 | **Better working environment** determines the country of choice participant intend to migrate. Yes = 1, No = 0. |
| 122 | intent_dest_factors_7 | **Professional advancement opportunities** determines the country of choice participant intend to migrate. Yes = 1, No = 0. |
| 123 | intent_dest_factors_8 | **Improved knowledge and skills** determines the country of choice participant intend to migrate. Yes = 1, No = 0. |
| 124 | intent_dest_factors_9 | **Research opportunities** determines the country of choice participant intend to migrate. Yes = 1, No = 0. |
| 125 | intent_dest_factors_10 | **Ease of finding a job abroad** determines the country of choice participant intend to migrate. Yes = 1, No = 0. |
| 126 | intent_dest_factors_11 | **Better job satisfaction** determines the country of choice participant intend to migrate. Yes = 1, No = 0. |
| 127 | intent_dest_factors_12 | **Professional status/prestige** determines the country of choice participant intend to migrate. Yes = 1, No = 0. |
| 128 | intent_dest_factors_13 | **Job security** determines the country of choice participant intend to migrate. Yes = 1, No = 0. |
| 129 | intent_dest_factors_14 | **No qualifying exams are required to practice** determines the country of choice participant intend to migrate. Yes = 1, No = 0. |
| 130 | intent_dest_factors_15 | **Better quality of life including psychological wellbeing** determines the country of choice participant intend to migrate. Yes = 1, No = 0. |
| 131 | intent_dest_factors_16 | **Peer Influence** determines the country of choice participant intend to migrate. Yes = 1, No = 0. |
| 132 | intent_dest_factors_17 | **Taste of something new** determines the country of choice participant intend to migrate. Yes = 1, No = 0. |
| 133 | intent_dest_factors_18 | **Family members abroad** determines the country of choice participant intend to migrate. Yes = 1, No = 0. |
| 134 | intent_dest_factors_19 | **Weather** determines the country of choice participant intend to migrate. Yes = 1, No = 0. |
| 135 | intent_dest_factors_20 | **Previous travel abroad for professional reasons** determines the country of choice participant intend to migrate. Yes = 1, No = 0. |
| 136 | intent_dest_factors_21 | **Social and cultural factors including language, cultural similarities, or personal freedom** determines the country of choice participant intend to migrate. Yes = 1, No = 0. |
| 137 | intent_dest_other | Indicate if the study participant considered migrating to other countries. The options are Yes or No. |
| 138 | intent_dest_other_list | Indicate which other countries the study participant considered migrating to. The options are multiple responses (countries). |
| 139 | f_push_factor | Indicate what factors discouraged the participant from migrating to the other countries they considered **(i.e., foreign push factors)**. Multiple response. |
| 140 | f_push_factor_1 | **Racism** was considered a foreign push factor by the study participant. Yes = 1, No = 0. |
| 141 | f_push_factor_2 | **Language barrier** was considered a foreign push factor by the study participant. Yes = 1, No = 0. |
| 142 | f_push_factor_3 | **Religion and culture** was considered a foreign push factor by the study participant. Yes = 1, No = 0. |
| 143 | f_push_factor_4 | **Qualifying examinations** was considered a foreign push factor by the study participant. Yes = 1, No = 0. |
| 144 | f_push_factor_5 | **Poor career prospects including a lack of information about career pathway** was considered a foreign push factor by the study participant. Yes = 1, No = 0. |
| 145 | f_push_factor_6 | **Poor working conditions including long working hours** was considered a foreign push factor by the study participant. Yes = 1, No = 0. |
| 146 | f_push_factor_7 | **Inadequate remuneration** was considered a foreign push factor by the study participant. Yes = 1, No = 0. |
| 147 | f_push_factor_8 | **Lack of professionalism and respect** was considered a foreign push factor by the study participant. Yes = 1, No = 0. |
| 148 | f_push_factor_9 | **Cost of migration/ financial barriers** was considered a foreign push factor by the study participant. Yes = 1, No = 0. |
| 149 | f_push_factor_10 | **Weather conditions** was considered a foreign push factor by the study participant. Yes = 1, No = 0. |
| 150 | f_push_factor_11 | **Poor quality of life including fractured family life** was considered a foreign push factor by the study participant. Yes = 1, No = 0. |
| 151 | f_push_factor_12 | **Lack of opportunities for family members including lack of job opportunities for spouse or educational opportunities for children** was considered a foreign push factor by the study participant. Yes = 1, No = 0. |
| 152 | f_push_factor_other | **Other factors not listed were** considered a foreign push factor by the study participant. Options were multiple responses. |
| 153 | return_migration | Indicate what factors will persuade the study participant who have already migrated to return to Nigeria. |
| 154 | l_pull_factor | Indicate what factors dissuaded the participant from migrating from Nigeria **(i.e., local pull factors)**. Multiple response |
| 155 | l_pull_factor_1 | **Bonds** dissuaded the participant from migrating from Nigeria. Yes = 1, No = 0. |
| 156 | l_pull_factor_2 | **Racism in other countries** dissuaded the participant from migrating from Nigeria. Yes = 1, No = 0. |
| 157 | l_pull_factor_3 | **Language barrier in other countries** dissuaded the participant from migrating from Nigeria. Yes = 1, No = 0. |
| 158 | l_pull_factor_4 | **Religion and culture of other countries** dissuaded the participant from migrating from Nigeria. Yes = 1, No = 0. |
| 159 | l_pull_factor_5 | **Good remuneration in Nigeria** dissuaded the participant from migrating from Nigeria. Yes = 1, No = 0. |
| 160 | l_pull_factor_6 | **Professional satisfaction including professional accomplishment in Nigeria** dissuaded the participant from migrating from Nigeria. Yes = 1, No = 0. |
| 161 | l_pull_factor_7 | **Opportunity for career advancement in Nigeria** dissuaded the participant from migrating from Nigeria. Yes = 1, No = 0. |
| 162 | l_pull_factor_8 | **Job security in Nigeria** dissuaded the participant from migrating from Nigeria. Yes = 1, No = 0. |
| 163 | l_pull_factor_9 | **Good working conditions in Nigeria** dissuaded the participant from migrating from Nigeria. Yes = 1, No = 0. |
| 164 | l_pull_factor_10 | **Qualifying examinations to practice in other countries** dissuaded the participant from migrating from Nigeria. Yes = 1, No = 0. |
| 165 | l_pull_factor_11 | **Poor career prospects including a lack of information about career pathway in other countries** dissuaded the participant from migrating from Nigeria. Yes = 1, No = 0. |
| 166 | l_pull_factor_12 | **Poor working conditions including long working hours in other countries** dissuaded the participant from migrating from Nigeria. Yes = 1, No = 0. |
| 167 | l_pull_factor_13 | **Inadequate remuneration in other countries** dissuaded the participant from migrating from Nigeria. Yes = 1, No = 0. |
| 168 | l_pull_factor_14 | **Family ties in Nigeria** dissuaded the participant from migrating from Nigeria. Yes = 1, No = 0. |
| 169 | l_pull_factor_15 | **Culture and social life in Nigeria** dissuaded the participant from migrating from Nigeria. Yes = 1, No = 0. |
| 170 | l_pull_factor_16 | **Desire to raise children in Nigeria** dissuaded the participant from migrating from Nigeria. Yes = 1, No = 0. |
| 171 | l_pull_factor_17 | **Religion in Nigeria** dissuaded the participant from migrating from Nigeria. Yes = 1, No = 0. |
| 172 | l_pull_factor_18 | **Advancing age** dissuaded the participant from migrating from Nigeria. Yes = 1, No = 0. |
| 173 | l_pull_factor_19 | **Lack of necessary resources to migrate out of Nigeria** dissuaded the participant from migrating from Nigeria. Yes = 1, No = 0. |
| 174 | l_pull_factor_20 | **Having financial security including ownership of business or properties in Nigeria** dissuaded the participant from migrating from Nigeria. Yes = 1, No = 0. |
| 175 | l_pull_factor_21 | **Patriotism including the desire to settle in Nigeria/optimism about the future in Nigeria** dissuaded the participant from migrating from Nigeria. Yes = 1, No = 0. |
| 176 | l_pull_factor_22 | **Weather conditions in other countries** dissuaded the participant from migrating from Nigeria. Yes = 1, No = 0. |
| 177 | l_pull_factor_23 | **Poor quality of life including fractured family life in other countries** dissuaded the participant from migrating from Nigeria. Yes = 1, No = 0. |
| 178 | l_pull_factor_24 | **Lack of opportunities for family members in other countries** dissuaded the participant from migrating from Nigeria. Yes = 1, No = 0. |
| 179 | return_migration_nig | Indicate if study participant already migrated from Nigeria but returned back. Options are Yes and No. |
| 180 | return_migr_nig_yr_out | Indicate which year participant migrated, before they returned |
| 181 | return_migr_nig_dest | Indicate which country participant migrated to, before the returned |
| 182 | return_migr_nig_yr_in | Indicate which year participant returned to Nigeria |
| 183 | return_migr_nig_factor | Indicate which factors influenced participant decision to leave their destination country and return to Nigeria |
| 184 | qual_yes_no | Indicate if participant is willing to be contacted for a qualitative focus group discussion. |
